# Proceedings of the Rock River Medical Society

**Published:** 1847-12

**Authors:** D. G. Clark

**Affiliations:** Secretary pro. tem.


ARTICLE VI.

Messrs. Editors:—The following is a brief abstract of the proceedings of the Rock River Medical Society, at its last session, which you are at liberty to publish if you please. Owing to the inclement weather and the bad state of the roads, the meeting was not quite so large as usual, but still the exercises throughout, were highly interesting, and the members manifested a noble zeal in sustaining the interests of the medical profession at the West. The hour of our meeting was too brief. Such meetings, with kindred spirits, who have common interests, are always too short. They are to the toil worn physician what the fragrant oasis is to the weary traveller.

Pursuant to adjournment, the Rock River Medical Society held its semi-annual meeting, at Beloit, W. T., 16th November, 1847.

The President being absent, A. Clark, M. D., the 1st. Vice President, took the chair, Dr. VanBrunt was appointed Vice President, pro. tem., and Dr. D. G. Clark, Secretary pro tem.

The meeting being duly organized, the following gentlemen, on motion, were admitted members of the Society, to wit: Drs. Moore, Williams, Davis, Hooker, Clark, and Lane.

A paper was then read by Dr. S. G. Armor, on the Symptomatology of Disease, as furnishing indications of cure.

Many interesting cases were then reported, and much valuable information elicited upon subjects connected with the interests of the profession at the West.

Dr. I. C. Goodhue offered the following resolution which was unanimously adopted by the Society.

*Resolved*, That this Society will hereafter diligently inquire into, and vigorously enforce the Constitution and By-Laws, against any member or members who may countenance or consult with imposters in medicine or surgery.

Dr. L. Clark then offered the following resolution, which was unanimously adopted :

*Resolved*, That in the opinion of this Society, the reforms suggested by the American Medical Association as to the preliminary education of Students, their time of preparatory study, the branches taught in medical schools, length of sessions thereof, &c., are loudly demanded, and meet our entire approbation.

The following resolution was then offered by Dr, Ames, and unanimously adopted:

*Resolved*, That this Society do now elect the number of delegates to which it is entitled in the American Medical Association, which will meet at Baltimore, May, 1848.

Whereupon, Drs. I. C. Goodhue, S. G. Armor, A. E. Ames, J. B. Nash, and A. Clark, were appointed said delegates.

On motion, the Society adjourned to meet at the First Congregational Church, at six and a half o'clock, p. m., at which time the Society and a large number of citizens, who had assembled agreeably to invitation, listened to eloquent and appropriate addresses from Drs. Goodhue and Armor.

At the close of these exercises, the citizens retiring, a full and free discussion was entered into in relation to the character, type, symptoms, and pathology of Typhus, Typhoid, and Periodical Fevers.

On motion, Dr. L. Clark was appointed to read an essay at the next annual meeting at Dixon, and Dr. Ames to deliver a lecture at the same time and place.

The Society, after returning their thanks to Drs. Goodhue and Armor, for their able and interesting addresses, adjourned to meet at Dixon, Illinois, on the third Tuesday in May next.

D. G. CLARK, *Secretary pro. tem.*

*Rock River Medical Society.*

